# Microbial metabolite deoxycholic acid controls *Clostridium perfringens*-induced chicken necrotic enteritis through attenuating inflammatory cyclooxygenase signaling

**DOI:** 10.1038/s41598-019-51104-0

**Published:** 2019-10-10

**Authors:** Hong Wang, Juan D. Latorre, Mohit Bansal, Mussie Abraha, Bilal Al-Rubaye, Guillermo Tellez-Isaias, Billy Hargis, Xiaolun Sun

**Affiliations:** Center of Excellence for Poultry Science, University of Arkansas, 1260W Maple St., Fayetteville, AR 727101 USA

**Keywords:** Animal physiology, Infection

## Abstract

Necrotic enteritis (NE) caused by *Clostridium perfringens* infection has reemerged as a prevalent poultry disease worldwide due to reduced usage of prophylactic antibiotics under consumer preferences and regulatory pressures. The lack of alternative antimicrobial strategies to control this disease is mainly due to limited insight into the relationship between NE pathogenesis, microbiome, and host responses. Here we showed that the microbial metabolic byproduct of secondary bile acid deoxycholic acid (DCA), at as low as 50 µM, inhibited 82.8% of *C. perfringens* growth in Tryptic Soy Broth (P < 0.05). Sequential *Eimeria maxima* and *C. perfringens* challenges significantly induced NE, severe intestinal inflammation, and body weight (BW) loss in broiler chickens. These negative effects were diminished (P < 0.05) by 1.5 g/kg DCA diet. At the cellular level, DCA alleviated NE-associated ileal epithelial death and significantly reduced lamina propria cell apoptosis. Interestingly, DCA reduced *C. perfringens* invasion into ileum (P < 0.05) without altering the bacterial ileal luminal colonization. Molecular analysis showed that DCA significantly reduced inflammatory mediators of *Infγ*, *Litaf*, *Il1β*, and *Mmp9* mRNA accumulation in ileal tissue. Mechanism studies revealed that *C. perfringens* induced (P < 0.05) elevated expression of inflammatory mediators of *Infγ*, *Litaf*, and *Ptgs2* (Cyclooxygenases-2 (COX-2) gene) in chicken splenocytes. Inhibiting the COX signaling by aspirin significantly attenuated INFγ-induced inflammatory response in the splenocytes. Consistent with the *in vitro* assay, chickens fed 0.12 g/kg aspirin diet protected the birds against NE-induced BW loss, ileal inflammation, and intestinal cell apoptosis. In conclusion, microbial metabolic product DCA prevents NE-induced BW loss and ileal inflammation through attenuating inflammatory response. These novel findings of microbiome protecting birds against NE provide new options on developing next generation antimicrobial alternatives against NE.

## Introduction

Antimicrobial resistance is one of the emerging challenges requiring immediate and sustainable counter-actions from agriculture to healthcare^1^. Increasing antimicrobial resistance has caused the emergence of multiple drug-resistant microbes or “superbugs”. Recently, a “superbug” of an *Escherichia coli* strain resistant to the last resort antibiotic, Colistin, was reported in USA^2^. Overuse of antimicrobial agents in medical and agricultural practice is contributing to exacerbating the episodes of emerging antimicrobial resistant microbes^1^. Withdrawing antimicrobials in chicken production, however, has caused new problems for the chicken industry by reducing production efficiency and increasing diseases, such as *Eimeria maxima*- and *Clostridium perfringens*-induced necrotic enteritis (NE)^3^. NE is a multi-factorial disease and has a significant economic impact to the chicken industry with annual loss at billions of dollars^4^. Despite no clinical NE outbreak worldwide, subclinical NE (cholangiohepatitis) incidences were doubled from 2013 to 2014 in South-Eastern Norway and were associated with reduced in-feed antimicrobial usage^5^. Clinical signs of acute NE include watery to bloody (dark) diarrhea, severe depression, decreased appetite, closed eyes, and ruffled feathers. Dissection of dead or severely ill birds shows that the intestine is often distended with gas, very friable, and contains a foul-smelling brown fluid, with clearly visible necrotic lesions^6^. At the cellular level, NE birds display intestinal inflammation with diffuse and coagulative necrosis of villi and crypts, infiltration of immune cells into lamina propria, and crypt abscesses^7,8^. Although progress has been made toward understanding risk factors influencing the outcome of NE such as *C. perfringens* virulence, coccidiosis, and feed^9^, few effective non-antimicrobial strategies are available.

The human and animal intestine harbors up to trillions of microbes and this intestinal microbiota regulates various host functions such as the intestinal barrier, nutrition and immune homeostasis^10–12^. The enteric microbiota regulates granulocytosis and neonatal response to *Escherichia coli K1* and *Klebsiella pneumoniae* sepsis^13^, suggesting the key role of the microbiota in protecting the host against systemic infection. At the gut level, fecal transplantation was reported decades ago to prevent *Salmonella infantis* chicken infection^14^. More recently, microbiota transplantation has shown tremendous success against recurrent human *Clostridium difficile* infection^15^ and *Clostridium scindens* metabolizing secondary bile acids have been shown to inhibit *C. difficile* infection^16^. Bile acids synthesized in the liver are released in the intestine and metabolized by gut microbiota into final forms of secondary bile acids^17^. Secondary bile acid of deoxycholic acid (DCA) is associated with a variety of human chronic diseases, such as obesity, diabetes, and colorectal cancer^18,19^. Recently, mouse anaerobes and their metabolic product DCA has been found to prevent and treat *Campylobacter jejuni*-induced intestinal inflammation in germ-free mice through attenuating host inflammatory signaling pathways^20^. However, whether bile acids play any role in NE pathogenesis is unknown.

NE is frequently concurrent with the episode of coccidiosis^21^. Coccidiosis is associated with strong immune response and intestinal inflammation in chickens^22,23^. Coccidia-induced intestinal inflammation may render a favorable environment for *C. perfringens* overgrowth, virulence expression, and invasion^21^. *C. perfringens* produces various toxins^24^ and induces hemolysis, epithelial barrier dysfunction, tissue necrosis and severe inflammation in non-chicken models^25,26^. Among inflammatory signaling pathways, cyclooxygenases (COX)-catalyzed prostanoids regulate various activities including cell proliferation, apoptosis and migration^27^, gastrointestinal secretion^28^, body temperature^29^, inflammation^30^, and pain sensation^31^. COX-1 and COX-3, translated from alternative splicing gene of *Ptgs1*, are constitutively expressed in intestinal cells and they maintain intestinal epithelial integrity. Inducible COX-2 (*Ptgs2*) activity is associated with various inflammatory diseases including inflammatory bowel disease^32^ and radiation-induced small bowel injury^33^. COX-2 increases gut barrier permeability and bacterial translocation across the intestinal barrier^34,35^. Paradoxically, COX-2 enhances inflammation resolution through prostaglandin D_2_^36^. Although non-selective COX inhibitor, aspirin, is used to prevent various chronic diseases, it inflicts intestinal inflammation to the healthy intestine^37^. However, the role of COX signaling on NE remains elusive.

Currently, limited knowledge is available on the relationship among the NE pathogenesis, the microbiome, and the host inflammatory response. Because DCA attenuates *C. jejuni*-induced intestinal inflammation^20^ and prevents *in vitro* growth of *C. difficile*^16^, a same genus member of *C. perfringens*, we hypothesized that the microbiota metabolic product DCA attenuated NE through inhibiting intestinal inflammation. We found that DCA decreased NE-induced intestinal inflammation, *C. perfringens* invasion, intestinal cell death, and body weight loss. Blocking the inflammatory COX signaling pathways by aspirin reduced NE-induced intestinal inflammation. These novel findings of microbiome DCA and COX inhibitor against NE offer new strategies to prevent and treat *C. perfringens*-induced diseases.

## Materials and Methods

### Bile acid inhibition of *C. perfringens* assay

Similar to bile acid inhibition assay on *Campylobacter jejuni* growth^38^, 10^3^ CFU *C. perfringens* was inoculated into 10 ml Tryptic Soy Broth (TSB) supplemented with 0.5% sodium thioglycollate, in the presence of taurocholic acid (TCA, 0.2 mM, final concentration), cholic acid (CA, 0.2 mM), or DCA (0, 0.01, 0.05, 0.1, or 0.2 mM), or 0, 0.2, or 1 mM of lithocholic acid (LCA) or ursodeoxycholic acid (UDCA). The different treatments of bacterial broth were cultured at 42 °C overnight (16–18 hours) under anaerobic conditions. The bacterial growth in tubes was observed for inhibition (clear broth) or no inhibition (cloudy broth). The bacterial growth was quantified by OD_600_ nm using a spectrophotometer (Nanodrop, Thermo Fisher).

### Chicken experiment

Animal experiments performed were in accordance with the Animal Research: Reporting of *In Vivo* Experiments (https://www.nc3rs.org.uk/arrive-guidelines). The experiments were approved by the Care and Use Committee of the University of Arkansas. Cohorts of thirteen zero-day-old broiler chicks per group were obtained from Cobb-Vantress Hatchery (Siloam Springs, AR). Chicks were neck-tagged and randomly allocated to floor pens with new pine shavings as litter in an environmentally controlled isolated room suitable for up to biosafety level (BSL) III animal experiments. The birds were provided with their respective diet and water *ad libitum*, and temperature was maintained at 34 °C for the first 5 days of age and was then gradually reduced until a temperature of 23 °C was achieved at day 26 days of age. The birds were fed a corn-soybean meal-based starter diet during 0–10 days of age and a grower diet during 11–26 days of age. The basal diet was formulated as described before^39^. Treatment diets were supplemented with 1.5 g/kg CA or DCA (all from Alfa Aesar). No antibiotics, coccidiostats or enzymes were added to the feed. *E. maxima* kindly provided by Dr. John Barta was M6 strain, which was a single oocyst-derived isolate at Ontario Veterinary College in 1973. The *E. maxima* was propagated in chickens as describe before^40^. The recovered oocysts were sporulated as previously described^41^. *C. perfringens* kindly donated from USDA-ARS, College Park, TX^42,43^ was confirmed alpha-toxin positive using a multiplex PCR assay^42,44^. An aliquot of frozen *C. perfringens* was grown in TSB plus sodium thioglycollate overnight for the NE challenge study, and was serially diluted and plated on Tryptic Soy Agar plus sodium thioglycolate for enumerating CFU. In previous experiments, birds infected with this aliquot of *C. perfringens* alone didn’t show any signs of NE and had comparable body weight gain to noninfected birds (data not showed). In current experiments, birds were infected with 20,000 sporulated *E. maxima* oocytes/bird at 18 days of age and 10^9^ CFU *C. perfringens*/bird at 23 and 24 days of age. Chicken body weight and feed intake were individually measured at 0, 18, 23, and 26 days of age. Bird health status was monitored daily after the pathogen infection. Four birds with average BW of the treatment group were sacrificed at 23 and 26 days of age. Because gross necrotic lesion was often observed in upper ileum in this NE model, the ileal tissue and digesta samples from all sacrificed birds were collected for RNA and DNA analysis. The ileal tissue of ~8 cm length was also Swiss-rolled for H&E staining and histopathology analysis. Images were acquired using a Nikon TS2 fluorescent microscope. Ileal inflammation was scored blindly using the H&E Swiss-roll slides (4 slides (birds)/treatment). Briefly, each Swiss-roll slide was divided into 4 areas and was scored. Total histopathological scores were then calculated by adding the four area scores. The following score scales used were based on previous ileitis^45,46^ and colitis scoring systems^47^: score 0: no inflammation, villi and crypt intact; score 1: small number infiltration cells in laminar propria of villi and crypts or villi minimally shortened; score 2: more extensive infiltration cells in laminar propria of villi and crypts, villi shortened >1/4 and edema, or crypt hyperplasia; score 3: pronounced infiltration cells in laminar propria of villi, crypts, submucosa, and muscularis, villi shortened >1/2 and edema, or crypt hyperplasia and regeneration; and score 4: necrosis, villus diffuse, ulcers, crypt abscesses, or transmural inflammation (may extend to serosa).

### Terminal deoxynucleotidyl transferase dUTP nick end labeling (TUNEL) assay

Cell apoptosis in intestinal tissue was visualized using TUNEL assay similar to described before^48^. Briefly, ileal tissue slides were deparaffinized with xylene bath for 3 times and then rehydrated with 100%, 95%, and 70% ethanol. The tissue was then incubated with TUNEL solution (5 µM Fluorescein-12-dUTP (Enzo Life Sciences), 10 µM dATP, 1 mM pH 7.6 Tris-HCl, 0.1 mM EDTA, 1U TdT enzyme (Promega)) at 37 °C for 90 min. The slides were counter-stained with DAPI for nucleus visualization. The fluorescent green apoptosis cells were evaluated and imaged using a Nikon TS2 fluorescent microscopy. The green dots in representative 3 areas per slide were counted as apoptosis cells and blue dots of nuclei were counted as total cells using ImageJ^49^ particle analysis and its plugin of Nikon ND2 reader. The results were showed as apoptosis cells per 1,000 total intestinal cells.

### Real time PCR of mRNA gene expression and *C. perfringens* quantification

Total RNA from ileal tissue or splenocytes was extracted using TRIzol as described before^20,50^. cDNA was prepared using M-MLV (NE Biolab). mRNA levels of proinflammatory genes were determined using SYBR Green PCR Master mix (Bio-Rad) on a Bio-Rad 384-well Real-Time PCR System and normalized to *Gapdh*. To quantify *C. perfringens* intestinal luminal colonization or tissue invasion, ileal digesta or tissue were weighed, bead-beaten, and extracted for DNA using phenol-chloroform method as described before^20^. *C. perfringens* in the digesta and tissue was quantified using specific *C. perfringens* 16S rDNA primers by the qPCR. The PCR reactions were performed according to the manufacturer’s recommendation. The following gene primers were used:

*Cp16S_*forward: 5′-CAACTTGGGTGCTGCATTCC-3′; *Cp16S_*reverse: 5′-GCCTCAGCGTCAGTTACAG-3′; *Mmp9*_forward: 5′-CCAAGATGTGCTCACCAAGA-3′; *Mmp9*_reverse: 5′-CCAATGCCCAACTTCTCAAT-3′; *Litaf_*forward: 5′-AGATGGGAAGGGAATGAACC; *Litaf_*reverse: 5′-GACGTGTCACGATCATCTGG-3′; *Il1β*_forward: 5′-GCATCAAGGGCTACAAGCTC-3′; *Il1β*_reverse: 5′-CAGGCGGTAGAAGATGAAGC-3′; *Infγ_*forward: 5′-AGCCGCACATCAAACACATA-3′; *Infγ_*reverse: 5′-TCCTTTTGAAACTCGGAGGA-3′; *Ptgs2*_forward: 5′-ACCAGCATTTCAACCTTTGC-3′; *Ptgs2*_reverse: 5′-CCAGGTTGCTGCTCTACTCC-3′; *Gapdh_*forward: 5′-GACGTGCAGCAGGAACACTA-3′; *Gapdh*_reverse: 5′-CTTGGACTTTGCCAGAGAGG-3′. Gene expression of fold change was calculated using ΔΔCt method^51^ and *Gapdh* as internal control.

### Fluorescence *in situ* hybridization (FISH)

*C. perfringens* at ileal tissue was visualized using FISH assay similarly to previously described^47^. Briefly, tissue slides were deparaffinized, hybridized with the FISH probe, washed, stained with DAPI, and imaged using a Nikon TS2 fluorescent microscope system. The FISH probe sequence of Cp85aa18: 5′-/Cy3/TGGTTGAATGATGATGCC-3′^52^ was used to probe the presence of *C. perfringens* similar to a previous report^50^. Briefly, deparaffinized, formalin-fixed 5 μm thick sections were incubated for 15 min in lysozyme (300,000 Units/ml lysozyme; Sigma-Aldrich) buffer (25 mM Tris pH 7.5, 10 mM EDTA, 585 mM sucrose, and 0.3 mg/ml sodium taurocholate) at room temperature and hybridized overnight at 46 °C in hybridization chambers with the oligonucleotide probe (final concentration of 5 ng/μl in a solution of 30 percent formamide, 0.9 M sodium chloride, 20 mM Tris pH 7.5, and 0.01% sodium dodecyl sulfate). Tissue sections were washed for 20 min at 48 °C in washing buffer (0.9 M NaCl, 20 mM Tris pH 7.2, 0.1% SDS, 20% Formamide, and 10% Dextran Sulfate) and once in distilled water for 10 seconds. The slides were stained with DAPI for 2 min and dried at room temperature, mounted with 50% glycerol. *C. perfringens* in intestinal tissues were visualized using a Nikon TS2 fluorescent microscopy and representative images of each treatment were presented in the Results section.

### *C. perfringens*-induced inflammatory response using primary splenocytes

Splenocytes were isolated similarly to described previously^53^. Briefly, chicken spleen was resected, homogenized into splenocytes using frosted glass slides, and pooled together in RPMI 1640 medium supplemented with 2% fetal bovine serum, 2 mM L-glutamine, 50 µM 2-mercaptoethanol. After lysed the red blood cells, the collected cells were plated at 2 × 10^6^ cells/well in 6-well plates. The cells were pre-treated with 1.2 mM aspirin for 45 min. Cells were then challenged with murine INFγ (1 μg/ml, Pepro Tech), chicken INFγ (1 μg/ml, IBI Scientific), or *C. perfringens* (multiplicity of infection 100). The cells were lysed in TRIzol (Invitrogen) for RNA isolation after 2 or 4 hours of cytokines or *C. perfringens* treatment, respectively.

### Dietary aspirin against NE

Birds fed diet supplemented with 0 and 0.12 g/kg aspirin were raised, infected, and sampled as abovementioned. Ileal histopathology images and scores were collected. To evaluate cell death, TUNEL assay was performed in the ileal slides of the birds and the apoptotic cells were quantified. The impact of aspirin on body weight gain was also measured.

### Statistical analysis

For *in vitro* assay of bile acids against *C. perfringens* growth, data were first analyzed by One-way ANOVA for significant difference and then Bonferroni’s multiple comparison test using Prism 5.0 software. For other data, differences between treatments were analyzed pairwise using the nonparametric Mann–Whitney *U* test performed using Prism 5.0 software. The specific pairwise comparisons were showed in Results section and Figures. Sample (individual birds or cell wells) numbers of body weight, histology, and other assays were listed in respective subsections of Methods section. Values are shown as mean of samples in the treatment ± standard error of the mean as indicated. Experiments were considered statistically significant if *P* values were < 0.05.

### Ethics approval and consent to participate

All animal protocols were approved by the Institutional Animal Care and Use Committee of the University of Arkansas at Fayetteville.

## Results

### DCA prevents *C*. *perfringens in vitro* growth

**Figure 1:**
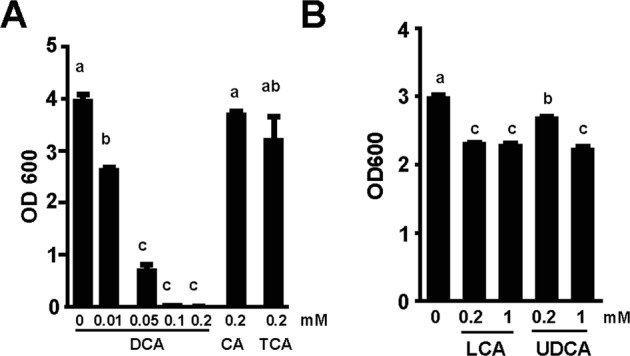
DCA inhibits *C. perfringens in vitro* growth. *C. perfringens* (10^3^ CFU) were inoculated into TSB supplemented with various concentrations of conjugated primary bile acid TCA and CA, secondary bile acids DCA, LCA, and UDCA. (**A**) OD_600_ reading of broth supplemented with DCA inhibiting *C. perfringens* growth. (**B**) OD_600_ reading of other secondary bile acids inhibiting *C. perfringens* growth at less efficiency compared to DCA. All graphs depict mean ± SEM. Different letters of a, b and c mean P < 0.05. Results are representative of 3 independent experiments.

Based on previous findings and current state of knowledge, we reasoned that DCA would prevent *C. perfringens* growth. To test this hypothesis, *in vitro* inhibition experiments were conducted, in which *C. perfringens* was inoculated in TSB with sodium thioglycollate under anaerobic condition. The TSB was also supplemented with various concentrations of bile acids, including conjugated primary bile acid TCA, primary bile acid CA, and secondary bile acid DCA. The results showed that DCA inhibited *C. perfringens* growth at 0.01 (−33.8%) and 0.05 mM (−82.8%, clear broth), respectively, compared to control, while TCA (−16.4%) and CA (−8.2%) didn’t prevent the bacterial growth (cloudy broth) even at 0.2 mM (Fig. 1A). We then examined whether other secondary bile acids were also bacteriostatic in TSB. Interestingly, *C. perfringens* growth was inhibited by lithocholic acid (LCA; −22.6 and −23.8%) and ursodeoxycholic acid (UDCA; −10.0 and −25.3%) at 0.2 and 1 mM, respectively (Fig. 1B) and it was far less effective compared to DCA.

### DCA prevents NE-induced intestinal inflammation in chicken ileum

**Figure 2:**
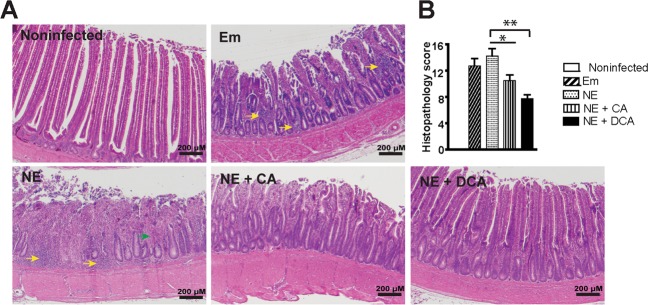
DCA attenuates NE-induced intestinal inflammation. Cohorts of thirteen broiler chicks were fed basal, 1.5 g/kg CA or DCA diets. The birds were infected with *E. maxima* at 18 days of age and *C. perfringens* at 23 and 24 days of age. Four birds per group were sacrificed and sampled at 26 days of age. (**A**) H&E staining showing representative intestinal histology images. (**B**) Quantification of histological intestinal damage score. Scale bar is 200 μm. All graphs depict mean ± SEM. *P < 0.05; **P < 0.01. NE + CA, NE birds fed CA diet; NE + DCA, NE birds fed DCA diet. Yellow arrows: immune cell infiltration; green arrow: fusion of villi and crypts. Results are representative of 3 independent experiments.

To further address whether DCA reduced coccidia *E. maxima*- and *C. perfringens*-induced NE in birds, broiler chicks were fed CA or DCA diet. Because coccidiosis and NE induce severe intestinal inflammation^7,8^, the impact of DCA on chicken NE was investigated. Upper ileum tissue was collected as Swiss-roll, processed with H&E staining, and performed histopathology analysis. Notably, *E. maxima* (Em) infection induced severe intestinal inflammation (ileitis) as seen by immune cell infiltration (yellow arrows) into lamina propria, crypt hyperplasia, and mild villus height shortening compared to uninfected birds (Fig. 2A). NE birds displayed worse ileitis as seen by necrosis and fusion of villi and crypt (Green arrow), massive immune cell infiltration, and severe villus shortening. In contrast, DCA diet dramatically attenuated NE-induced ileitis and histopathology score (Fig. 2B), while CA diet also reduced NE-induced ileitis and histopathology score.

### DCA attenuates NE-induced intestinal cell necrosis and apoptosis

**Figure 3:**
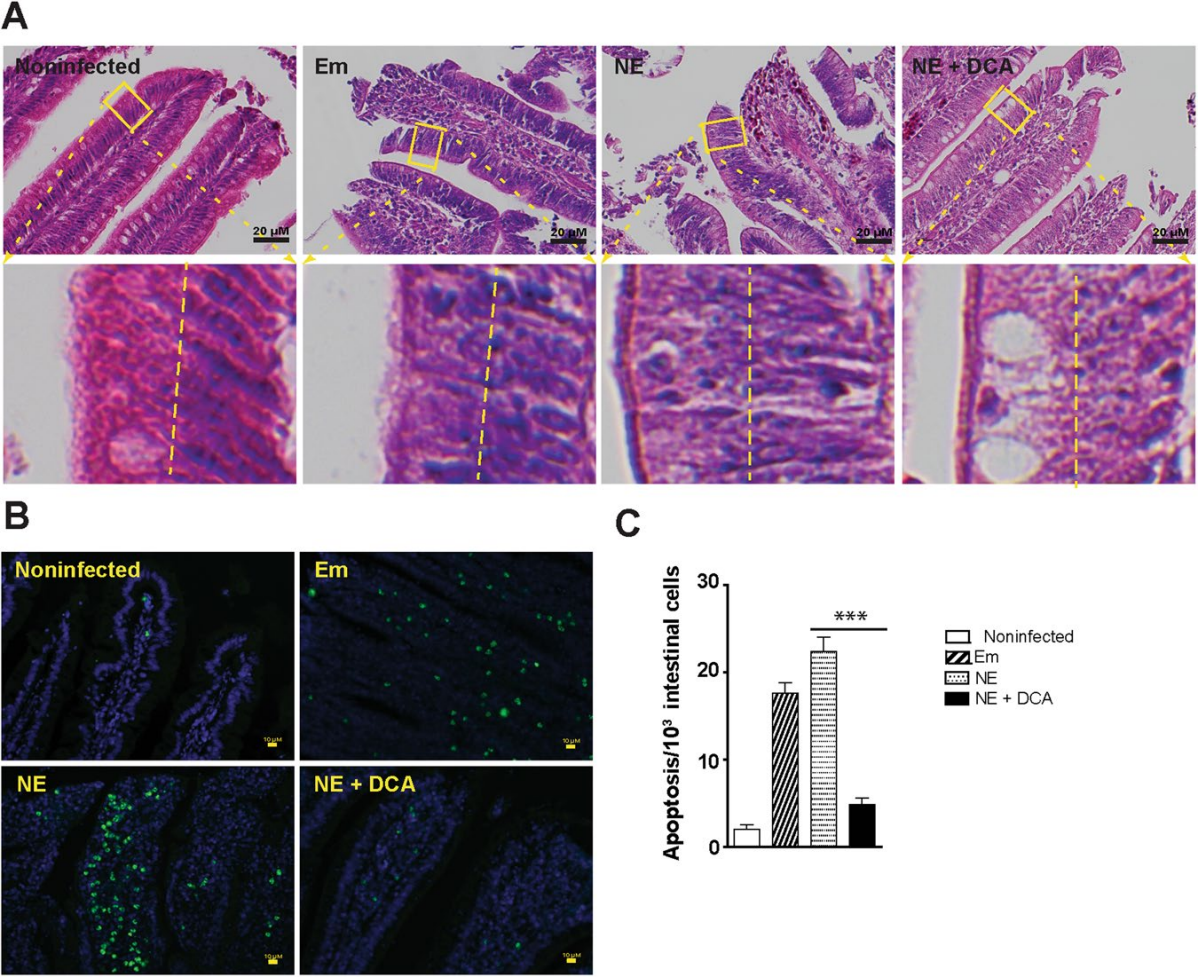
DCA attenuates NE-induced intestinal cell death and apoptosis. Cohorts of broiler chicks were fed different diets, infected, and sampled as in Fig. 2. (**A**) Representative intestinal cell death (deviated nuclei) using H&E staining. (**B**) Representative villus cell apoptosis (green) using TUNEL assay. (**C**) Quantified apoptotic cells using ImageJ. Scale bars are 20 μm (**A**) and 10 μm (**B**). NE + DCA, NE birds fed DCA diet. All graphs depict mean ± SEM. ***P < 0.001. Results are representative of 3 independent experiments.

Because inflammation often induces cell death^54^, it was then sought to examine whether cell death was relevant in DCA attenuating NE-induced ileitis. Since it was difficult to find reliable chicken antibodies to detect apoptosis or necrosis in chicken histology slides, we first resorted to classical histological analysis under high magnification. Consistently, the epithelial nuclei (dark blue) in heathy control bird villi were distributed close to the basal membrane (at the right side of the yellow dash line, Fig. 3A lower left panel). In contrast, the nuclei in inflamed villi epithelial cells of Em and NE birds were scattered from basal to the apical membranes, indicating epithelial cell death in villi of those birds. As a contrast, the DCA diet prevented epithelial cell nucleus translocation to apical side, suggesting cell death reduction. To further characterize the villus cell death, TUNEL assay was used, which detects later stage of cell apoptosis. Consistent with histopathology results, coccidiosis and NE induced scattered (Em birds) or concentrated (NE birds) apoptosis cells (green dots) in villus lamina propria, while cellular apoptosis was attenuated in the DCA treatment birds (Fig. 3B). To quantify the apoptosis, ImageJ was used to count apoptosis cells (TUNEL, green) and total cells (DAPI, blue). Consistent with histopathology results, DCA reduced NE-induced cell apoptosis (Fig. 3C).

### DCA reduces *C. perfringens* invasion, NE-induced inflammatory response, and productivity loss

**Figure 4:**
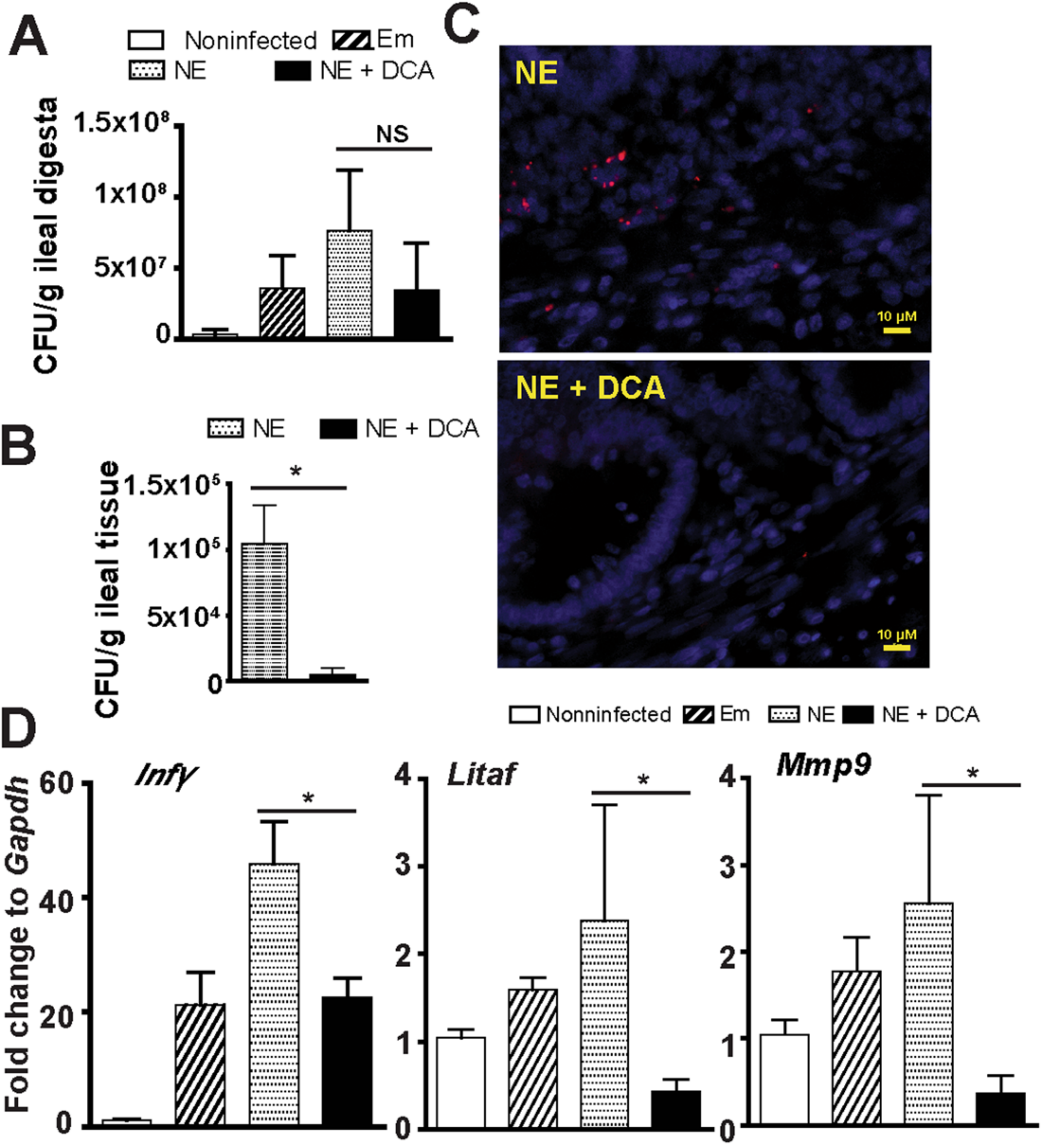
DCA reduces *C. perfringens* invasion and inflammatory response. Chickens were fed different diets, infected, and sampled as in Fig. 2. (**A**) Luminal *C. perfringens* colonization level quantified by 16 S rDNA real-time PCR. (**B**) *C. perfringens* invasion into intestinal tissue quantified by 16 S RNA real-time PCR. (**C**) Presence of *C. perfringens* (red dots) in ileal sections of NE and NE + DCA birds, detected using fluorescence *in situ* hybridization (FISH) assay. (**D**) Ileal *Infγ*, *Litaf* (*Tnfα*), and *Mmp9* mRNA qPCR fold change relative to uninfected birds and normalized to *Gapdh*. Scale bar is 10 μm. NE + DCA, NE birds fed DCA diet. All graphs depict mean ± SEM. NS, not significant; *P < 0.05. Results are representative of 3 independent experiments.

Given DCA inhibited *C. perfringens* growth *in vitro*, it was logic to reason that DCA might also reduce *C. perfringens* intestinal overgrowth in NE birds. To examine this possibility, *C. perfringens* colonization level was measured in the intestinal lumen using real-time PCR of *C. perfringens* 16S rDNA. Surprisingly, ileal luminal *C. perfringens* colonization in DCA birds was not significantly different from NE control birds (Fig. 4A), while bird histopathology was distinct between the two groups of birds. We then reasoned that the pathogen invasion into tissue was the main driving factors of NE pathogenesis, but not the pathogen luminal colonization level. To quantify the bacterial invasion, total DNA in ileal tissue was isolated and *C. perfringens* was measured using real time PCR. DCA attenuated more than 95% of *C. perfringens* invasion into ileal tissue (Fig. 4B). To have a better overview of the bacterial tissue invasion distribution, we used a fluorescence *in situ* hybridization (FISH) technique. We found that while *C. perfringens* was present deeply in the inflamed villus and crypt lamina propria of NE control birds, the bacterium was barely detectable in the ileal tissue of DCA birds (Fig. 4C). Because DCA reduced *C. perfringens* invasion and intestinal inflammation, the impact of DCA on various proinflammatory mediators was evaluated in ileal tissue using Real-Time PCR. *C. perfringens* induced inflammatory *Infγ*, *Litaf* (*Tnfα*), and *Mmp9* mRNA accumulation in chicken ileal tissue, an effect attenuated by 51, 82, and 93%, respectively, in DCA fed chickens (Fig. 4D).

**Figure 5:**
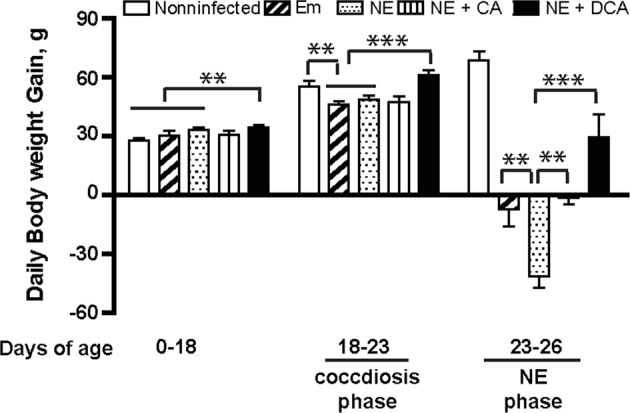
DCA attenuates NE-induced productivity loss. Cohorts of thirteen broiler chicks were fed different diets and infected as in Fig. 2. Bird body weight gain was measured at 18 (13 birds/group), 23 (13 birds/group), and 26 (8–9 birds/group) days of age. Showed was daily periodic body weight gain. NE + DCA, NE birds fed DCA diet. All graphs depict mean ± SEM. **P < 0.01; ***P < 0.001. Results are representative of 3 independent experiments.

Body weight (BW) is one of important productivity parameters for meat chickens and reflects collective response during NE. The productivity results showed that DCA (solid black bar) but not CA (vertical-line bar) diet promoted bird daily BW gain during 0–18 days of age compared to birds fed control diets (open, tilted line, and dotted bars, Fig. 5). BW gain was reduced in birds infected with *E. maxima* (Em) (tilted line and dotted bars) during 18–23 days of age (coccidiosis phase) compared to noninfected birds (open bar). Subsequent *C. perfringens* infection reduced NE control birds (dotted bar) BW gain during 23–26 days of age (NE phase) compared to Em birds (tilted line bar). Consistent with histopathology analysis, DCA prevented productivity loss at coccidiosis and NE phases compared to the NE control birds. Interestingly, the primary bile acid CA diet attenuated body weight loss at NE phase but failed at coccidiosis phase compared to the NE control birds.

### COX inhibitor aspirin alleviates *C. perfringens*-induced inflammatory response in splenocytes

**Figure 6:**
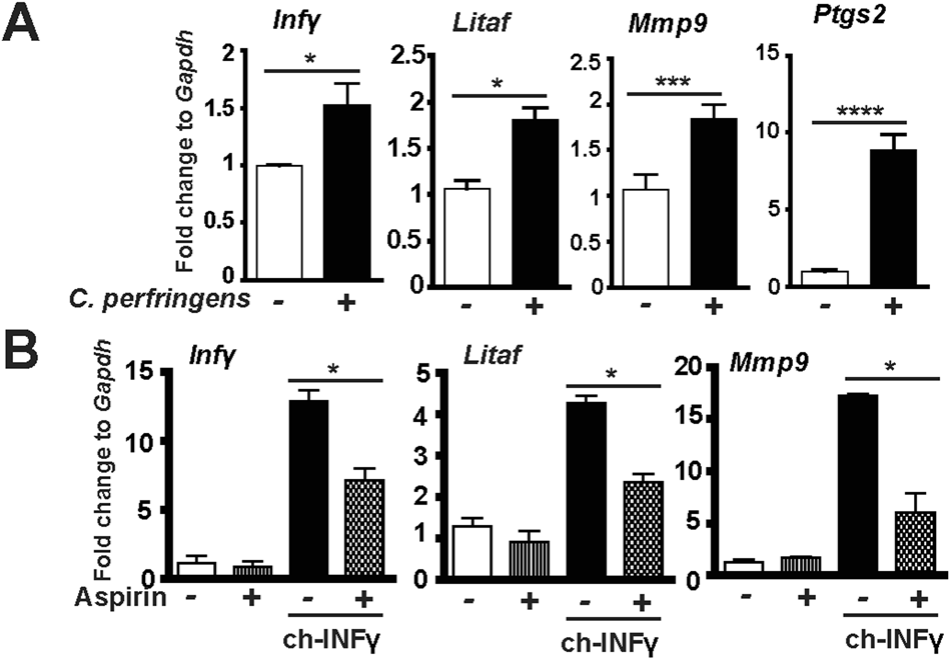
COX inhibitor aspirin alleviates *C. perfringens*-induced inflammatory response in chicken splenocytes. Splenocytes isolated from broiler chickens were infected with *C. perfringens* (MOI 100) for 4 hr or stimulated with chicken INFγ (1 μg/ml) for 2 hours in the presence of 1.2 mM aspirin. RNA was extracted, reverse-transcribed, and quantified using a Bio-Rad 384 PCR platform. (**A**) *Infγ*, *Litaf*, *Mmp9*, and *Ptgs2* mRNA fold change normalized to *Gapdh*. (**B**) Gene expression fold change in the presence of chicken INFγ (ch-INFγ) and aspirin. All graphs depict mean ± SEM. *P < 0.05; ***P < 0.001. Results are representative of 2 independent experiments.

**Figure 7:**
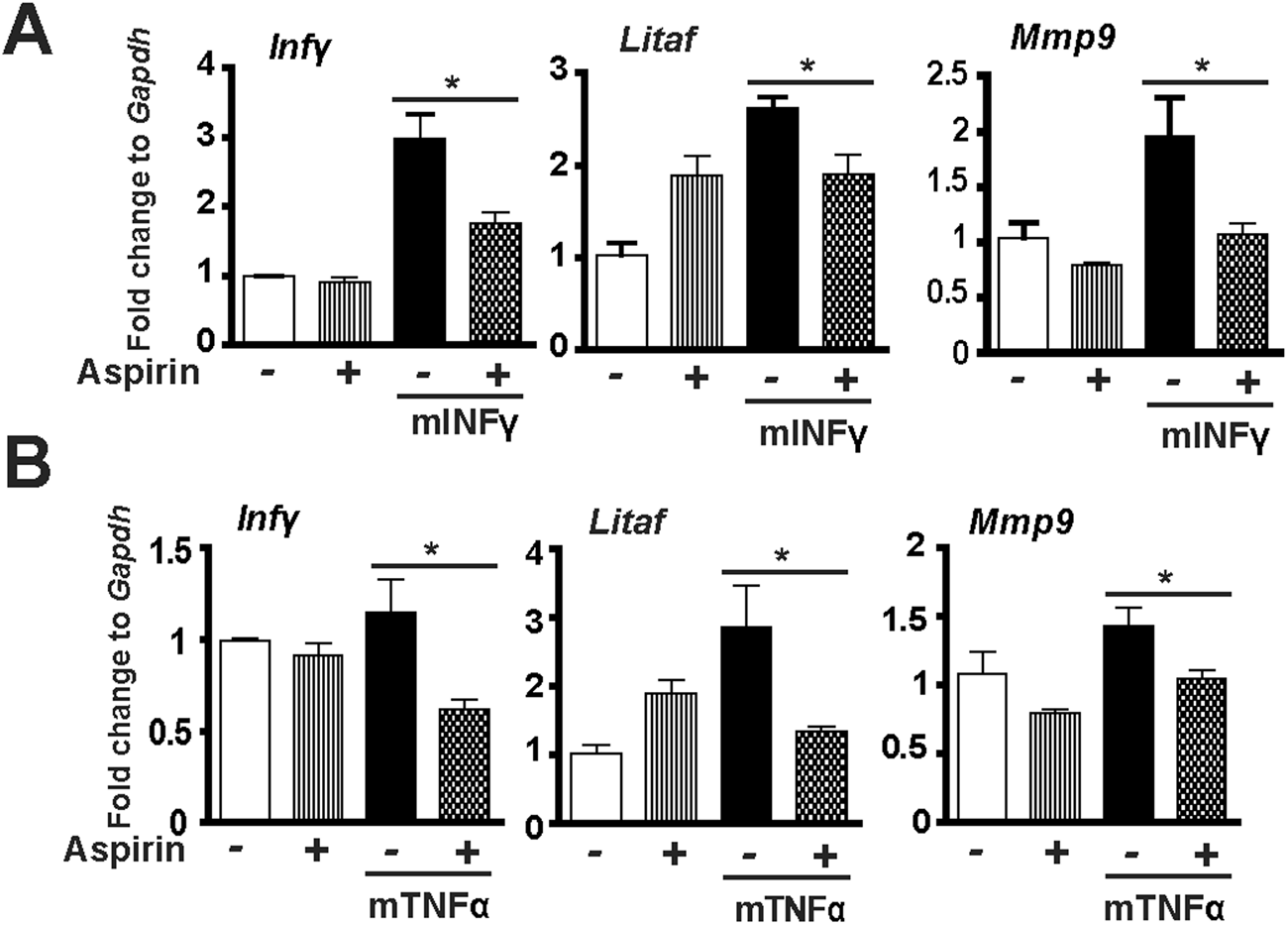
COX inhibitor aspirin alleviates murine cytokine-induced inflammatory response in chicken splenocytes. Splenocytes isolated from broiler chickens were stimulated with murine mINFγ (1 μg/ml) or mTNFα (5 ng/ml) for 2 hr in the presence of 1.2 mM aspirin. RNA was extracted, reverse-transcribed, and quantified using a Bio-Rad 384 PCR platform. (**A**) *Infγ*, *Litaf*, and *Mmp9* mRNA fold change normalized to *Gapdh* in the presence of mINFγ. (**B**) *Infγ*, *Litaf*, and *Mmp9* mRNA fold change normalized to *Gapdh* in the presence of mTNFα. All graphs depict mean ± SEM. *P < 0.05. Results are representative of 2 independent experiments.

Inflammatory events shape intestinal diseases and targeting the inflammatory response attenuates disease progress such as in Inflammatory Bowel Disease^55^ and campylobacteriosis^53^. To dissect how the host inflammatory response is involved in NE-induced ileitis, a primary chicken splenocyte cell culture system was then used similarly to previous report^47^. After isolation from chickens, the splenocytes were infected with *C. perfringens* (MOI 100) for 4 hours. The results showed that *C. perfringens* increased inflammatory mediators of *Infγ*, *Litaf* (*Tnfα*), *Mmp9* and *Ptgs2* (protein COX-2) mRNA accumulation by 1.54, 1.69, 1.72 and 8.65 folds, respectively, compared to uninfected splenocytes (Fig. 6A). Because COX-2 is an important mediator in the inflammatory response^32^, COX inhibitor aspirin was then used in *C. perfringens*-infected chicken splenocytes. Interestingly, aspirin failed to reduce *C. perfringens*-induced inflammatory gene expression (data not shown). We then reasoned that COX signaling acted on *C. perfringens*-induced inflammatory cytokines. Inflammatory cytokine of recombinant chicken INFγ (ch-INFγ) was then used to challenge splenocytes in the presence of aspirin. Aspirin reduced ch-INFγ-induced inflammatory gene expression of *Infγ*, *Litaf* (*Tnfα*), and *Mmp9* by 44, 45, and 65%, respectively (Fig. 6B). Because no chicken TNFα was available, murine INFγ (mINFγ) and mTNFα were used. Consistently, aspirin also reduced murine INFγ (mINFγ)-induced inflammatory gene expression of *Infγ*, *Litaf*, and *Mmp9* by 41, 27, and 45%, respectively (Fig. 7A). Similarly, aspirin reduced mTNFα-induced inflammatory gene expression of *Infγ*, *Litaf*, and *Mmp9* by 49, 53, and 27%, respectively (Fig. 7B). These data indicate that *C. perfringens* induces inflammatory cytokines and COX-2 while blocking COX signaling by aspirin reduces inflammatory cytokine-induced responses, suggesting that aspirin poses protection potential against NE detrimental inflammatory response.

### Aspirin attenuates NE-induced ileitis, intestinal cell apoptosis, and productivity loss

**Figure 8:**
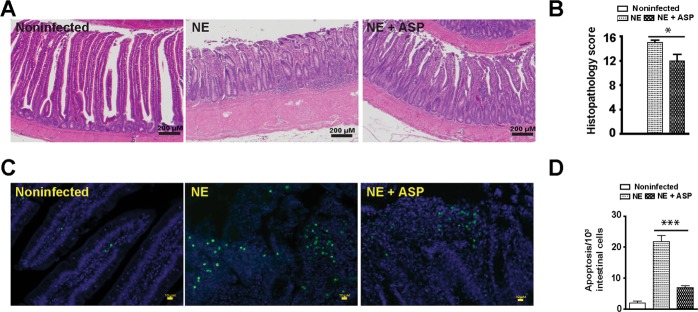
Aspirin attenuates NE-induced intestinal inflammation and apoptosis. Cohorts of thirteen chickens were fed basal and 0.12 g/kg aspirin diet. The birds were infected as in Fig. 2 and four birds per group were sampled at 26 days of age. (**A**) H&E staining showing representative intestinal histology images. (**B**) Quantification of histological intestinal damage score. (**C**) Representative cell apoptosis (green) using TUNEL assay. (**D**) Quantification of apoptotic cells. Scale bars are 200 μm in panel A and 10 μm in panel C. NE + ASP, NE birds fed aspirin diet. All graphs depict mean ± SEM. *P < 0.05; ***P < 0.001. Results are representative of 2 independent experiments.

**Figure 9:**
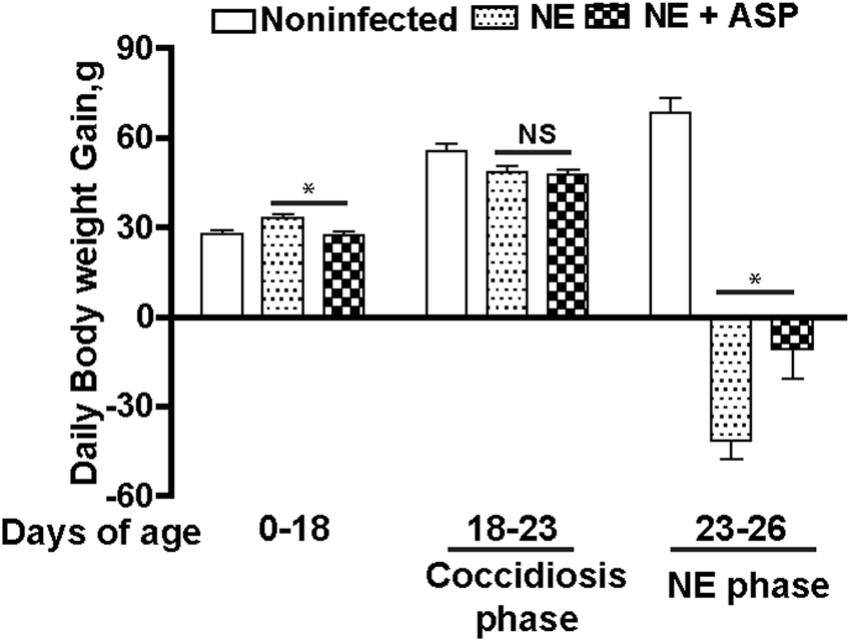
Aspirin attenuates NE-induced productivity loss. Cohorts of 13 broiler chicks were fed different diets and infected as in Fig. 6. Bird body weight gain was measured at 18 (13 birds/group), 23 (13 birds/group), and 26 (8 birds/group) days of age. Showed was daily periodic body weight gain. NE + ASP, NE birds fed aspirin diet. All graphs depict mean ± SEM. NS, not significant; *P < 0.01; Results are representative of 2 independent experiments.

To functionally assess the protective effect of aspirin against NE-induced ileitis, broiler chickens fed with aspirin diet (ASP) were infected with *E. maxima* and *C. perfringens* as describe before. Ileal tissues were collected and histopathology analysis was performed to assess NE. Consistently, NE birds had severe ileal necrosis with immune cell infiltration and villus shortening (Fig. 8A). Notably, ASP attenuated NE-induced intestinal inflammation and histopathological score (Fig. 8A,B). At cellular level, ASP reduced NE-induced immune cell apoptosis in villus lamina propria (Fig. 8C,D). On growth performance, ASP birds grew slower compared to control diet birds during 0–18 days of age (Fig. 9). This is because aspirin inhibits all COX isoforms and COX-1 and -3 are important for intestinal homeostasis and growth. Notably, ASP attenuated NE-induced BW loss by 60% during NE phase of 23–26 days of age, while no difference between ASP and NE birds during coccidiosis phase of 18–23 days of age.

## Discussion

Although NE has reemerged as a prevalent chicken disease worldwide in the antimicrobial free era^5^, the lack of comprehensive molecular mechanism insight into NE severely hinders the development of antimicrobial alternatives to control this disease^56^. Many virulence factors of *Eimeria* and *C. perfringens* are identified but few findings are effective to control NE in chicken production^9,57^, suggesting that important players/factors in NE pathogenesis were overlooked, such as microbiome and host response. We reported here that microbiota metabolic product DCA attenuated chicken NE by reducing chicken inflammatory COX signaling pathways. These new findings offer approaches for exploring novel antimicrobial alternatives to control *C. perfringens*-induced diseases.

It is a relatively new concept to manipulate microbiota and its metabolic products against infectious diseases. Fecal transplantation was used in chickens decades ago to prevent *S. infantis* infection^14^. Microbiome plays an important role in susceptibility to human *C. difficile* infection^58^. Anaerobe *C. scindens*-transformed secondary bile acids prevent *C. difficile* germination and growth^16^. LCA and DCA but not primary bile acid CA inhibit *C. difficile* vegetable growth and toxin production^16,59^. However, whether secondary bile acids prevent or treat *C. difficile* infection in human or animal models is still unknown. It has been recently found that orally gavaging DCA attenuated *C. jejuni*-induced intestinal inflammation in germ-free mice^20^. Based on the knowledge, we then reasoned that DCA might prevent *E. maxima*- and *C. perfringens*-induced chicken NE. Indeed, dietary DCA prevented NE and its associated productivity loss. The reduction of ileitis was coupled with reduced *C. perfringens* intestinal tissue invasion and intestinal inflammation and cell death. Intriguingly, DCA failed to reduce *C. perfringens* ileal luminal colonization, suggesting that the mechanism of DCA action is independent of intestinal luminal colonization exclusion and is possibly through modulating other factors such as inflammation.

At the cellular level, the intestinal tract of NE-inflicted birds displays severe small intestinal inflammation, showing massive immune cells infiltration into lamina propria, villus breakdown, and crypt hyperplasia^60,61^. Inflammation often induces cell death^54^. Normal intestinal epithelial cells have polarity^62^ and their nuclei are located toward the basal membrane^63^, while stressed dying (apoptosis or necrosis) cells lose polarity^64^ and their nuclei disperse from basal to apical membranes^65^. Intestinal inflammation is also critical to clear invaded microbes and to resolve inflammation, while overzealous inflammation causes more bacterial invasion and further collateral damage and inflammation^66^. Infectious bacteria often hijack the inflammatory pathways to gain survival and invasion advantage. For example, *Salmonella* Typhimurium induces extensive inflammation in mouse intestine and thrives on the inflammation^67^. Interestingly, *S*. Typhimurium infection causes immunosuppressive effect in neonatal chickens because of lymphocyte depletion^68^. Unlike *S*. Typhimurium infection in neonatal birds, coccidia infection induces strong immune response and intestinal inflammation in chickens^22,23^. Furthermore, NE birds suffered more intestinal inflammation compared to Em birds in current study. Consistent with the “over-inflammation” model, NE birds with severe intestinal inflammation showed extensive immune cell infiltration, increased inflammatory cytokine gene expression, and epithelial cell hyperplasia and death (dispersed nuclei and apoptosis). Conversely, DCA attenuated the intestinal inflammation and inflammatory cytokines and improved the growth performance and reduced the NE pathology. Consistent with the reasoning of anti-inflammation reducing NE, blocking inflammatory COX-2 signaling pathway by aspirin alleviated intestinal inflammation, villus apoptosis and NE-induced BW loss. These findings indicate that DCA attenuates NE through decreasing inflammatory signaling pathways.

Different strains of *C. perfringens* produce a variety of toxins including alpha (CPA), beta (CPB), epsilon (ETX), iota (ITX), enterotoxin (CPE), necrotic enteritis B-like toxin (NetB), and others^69^. Among them, researchers have reported that NetB but not CPA induced NE in chickens^70^. However, NE doesn’t have strong association with NetB positive *C. perfringens* in US^71^. In our NE model, no difference of *NetB* gene expression in ileal digesta between healthy control and NE birds (data not shown) suggests limited role of the toxin. Generally, *C. perfringens* toxins induce cell death of apoptosis, necrosis, necroptosis, and pyroptosis in the presence of extracellular calcium influx^69^. Higher dietary calcium increases chicken mortality and reduces growth performance in coccidiosis-induced NE model^72^, suggesting the important role of toxin-induced and calcium-mediated cell death in NE. The reduction of intestinal cell death by DCA and aspirin suggests possible toxin involvement in our NE model. Future work on *C. perfringens* toxins, DCA, and inflammatory response will be conducted.

In contrast to the accumulating evidence of bile acids against infectious enteritis in recent studies^16,20^, the microbial-derived metabolites have long been implicated in chronic diseases^18^. Plasm bile acids is correlated with increased body mass index of obese patients^73^. Plasma chenodeoxycholic acid, CA and DCA concentrations are higher in type 2 diabetes patients compared to healthy subjects^74^. Bile acid promoting fatty acid digestion and absorption may play important role in the diseases. Furthermore, DCA and LCA in the colonic contents are increased in humans consuming a high fat diet^75^. The increase of the two bile acids in feces is associated with elevated incidence of colorectal cancer^76^. DCA damages genomic DNA by oxidation^77^ and deficiency in base excision repair of oxidative DNA damage is linked to increased risk of intestinal tumors in mice^78^. Future work is needed to investigate how DCA induces the chronic diseases but attenuates the infectious enteritis.

Altogether, these data reveal that the microbial metabolic product secondary bile acid DCA attenuates NE, through reducing NE-induced host inflammatory response. These findings highlight the importance of elucidating the molecular relationship between infectious pathogen, microbiome, and host response. These discoveries could be applied to control NE and other intestinal diseases targeting microbiome and host inflammatory response.

## Data Availability

Data sharing is not applicable to this article as no datasets were generated or analyzed during the current study.
